# Treinamento Muscular Inspiratório em Diferentes Intensidades na Insuficiência Cardíaca: Há Diferenças nas Alterações Hemodinâmicas Centrais?

**DOI:** 10.36660/abc.20200162

**Published:** 2020-05-12

**Authors:** Lucas Helal, Filipe Ferrari

**Affiliations:** 1 Universidade do Extremo Sul Catarinense Criciúma SC Brasil Universidade do Extremo Sul Catarinense - UNESC, Criciúma, SC - Brasil; 2 Programa de Pós-Graduação em Cardiologia e Ciências Cardiovasculares Hospital de Clínicas de Porto Alegre Universidade Federal do Rio Grande do Sul Porto Alegre RS Brasil Programa de Pós-Graduação em Cardiologia e Ciências Cardiovasculares, Hospital de Clínicas de Porto Alegre (HCPA), Universidade Federal do Rio Grande do Sul, Porto Alegre, RS - Brasil; 3 Centre for Journalology Ottawa Hospital Research Institute Ottawa Canada Centre for Journalology, Ottawa Hospital Research Institute, Ottawa - Canada; 4 Grupo de Pesquisa em Cardiologia do Exercício Hospital de Clínicas de Porto Alegre Universidade Federal do Rio Grande do Sul Porto Alegre RS Brasil Grupo de Pesquisa em Cardiologia do Exercício (CardioEx), Hospital de Clínicas de Porto Alegre, Universidade Federal do Rio Grande do Sul, Porto Alegre, RS - Brasil

**Keywords:** Insuficiência Cardíaca, Pressões Respiratórias Máximas, Frequência Cardíaca, Volume Sistólico, Débito Cardíaco, Monitoração Hemodinâmica/métodos

A insuficiência cardíaca (IC) é uma entidade complexa e geralmente com prognóstico reservado, apontada como uma doença cardiovascular de extrema relevância devido à sua crescente incidência, prevalência e alta morbimortalidade associadas.^1^ Estima-se que a sua prevalência esteja variando entre 1% a 2% nos países desenvolvidos, atingindo >10% nas pessoas acima de 70 anos de idade;^2^ ademais, é considerada a principal causa cardiovascular de internação em indivíduos acima de 60 anos.^3^

Entre os principais sintomas característicos da IC, destacam-se dispneia e fadiga, os quais estão intimamente associados ao comprometimento da capacidade funcional e, consequentemente, da qualidade de vida desses indivíduos.^4^ O treinamento físico, por sua vez, ganhou um espaço de destaque nas últimas décadas, com o intuito de melhoria deste cenário, embasado por um crescente número de comprovações sólidas; sendo assim, ele tem a capacidade de impactar favoravelmente no ganho da capacidade funcional e qualidade de vida dos pacientes com IC.^5 , 6^ Dentre os diversos tipos de treinamento físico direcionados a esta população, o treinamento muscular inspiratório (TMI) é conhecido por sua fácil aplicabilidade e potenciais benefícios neste cenário.

## Treinamento muscular inspiratório na insuficiência cardíaca

Há evidências robustas sugerindo que a fraqueza dos músculos inspiratórios é um dos principais fatores que levam à baixa tolerância ao exercício nos pacientes com IC.^7 , 8^ De fato, ensaios clínicos randomizados comprovaram inúmeros benefícios do TMI em portadores desta síndrome, a citar: melhora significativa na eficiência na captação do oxigênio,^9^ capacidade funcional e escores de qualidade de vida.^10^ Estes resultados foram confirmados por metanálises, como aquela conduzida por Smart et al.^11^ Quando comparados ao grupo controle, os pacientes submetidos a TMI alcançaram uma melhora importante no consumo máximo de oxigênio (VO_2_máx): 1,83 ml.kg^-1^.min^-1^ (IC95%, 1,33 para 2,32 ml.kg^-1^.min^-1^, p <0,00001), bem como no teste de caminhada de 6 minutos: 34,35 m (IC95%, 22,45 para 46,24 m, p <0,00001). Por sua vez, a força muscular inspiratória parece ter uma correlação significativa com o VO_2_máx, o qual é um preditor independente de sobrevida nos indivíduos com IC.^12^ O TMI, deve, portanto, ser parte integrante dos cuidados destes pacientes sempre que possível.

Nesta edição dos Arquivos Brasileiros de Cardiologia, um ensaio clínico randomizado, placebo e controlado^13^ avaliou as implicações de uma sessão aguda de diferentes intensidades de TMI sobre a resposta hemodinâmica central (RHC) de indivíduos com IC, utilizando um método não invasivo de monitorização. Para tal, foram incluídos 20 pacientes com fração de ejeção reduzida (37,2% ± 6,3%), idade média de 65 anos, e na grande maioria em classe funcional II da *New York Heart Association* (NYHA). O protocolo do TMI se resumiu em 3 sessões durante 15 minutos cada. Todos os participantes realizaram o treinamento com intensidade em 30% e 60% da pressão inspiratória máxima (PImáx), além de intervenção *sham* (placebo), com *washout* de 1 hora entre elas. Foi observado que a RHC se comportou de forma heterogênea entre as intensidades. Por exemplo, houve um aumento da frequência cardíaca com as intensidades de 30% e 60% da PImáx (64 ± 15 para 69 ± 15 batimentos por minuto; e 67 ± 14 para 73 ± 14 batimentos por minuto, respectivamente). Em relação ao volume sistólico, houve tendência à redução com a carga de 30% da PImáx (73 ± 26 ml para 64 ± 20 ml). Já o débito cardíaco aumentou apenas no grupo de maior intensidade (4,6 ± 1,5 l/min para 5,3±1,7 l/min), comportamento que foi semelhante em relação à resposta da pressão arterial sistólica. De fato, o aumento do débito cardíaco observado na maior intensidade aplicada pode ser explicado, parcialmente, pelo aumento da frequência cardíaca neste grupo. Estes achados não devem ser ignorados, uma vez que os pacientes com IC tendem a apresentar fluxo sanguíneo prejudicado para os músculos em atividade, secundário às reduções do débito cardíaco e da capacidade vasodilatadora periférica. Estas alterações são prejudiciais, causando importante intolerância ao esforço, estando associadas à capacidade vasodilatadora reduzida e aumento da estimulação simpática comuns nesses indivíduos.^14^

Recentemente, através de uma revisão sistemática com metanálise, nosso grupo mostrou que o TMI de alta intensidade (≥ 60% da PImáx) pode ser uma estratégia eficiente para melhorar a capacidade funcional e a força muscular inspiratória desta mesma classe de pacientes (leia-se: IC e fração de ejeção reduzida).^15^ Por sua vez, acreditamos que o estudo em questão^13^ adiciona importantes conhecimentos à literatura, mostrando diferenças nas repercussões hemodinâmicas observadas no TMI em diferentes intensidades, uma área pouco explorada até o momento. Estes achados podem abrir novos horizontes e perspectivas, influenciando a realização de maiores pesquisas explorando as respostas hemodinâmicas do TMI em pacientes com IC. A falta de uma estreita correlação entre a hemodinâmica central (como hiperativação do sistema nervoso simpático) e a tolerância ao exercício fortalecem a importância dos resultados deste estudo.
