# Supplementary material for: Treinamento Muscular Inspiratório em Diferentes Intensidades na Insuficiência Cardíaca: Há Diferenças nas Alterações Hemodinâmicas Centrais?
Source: Arq Bras Cardiol. 2020 May 12;114(4):664–5. [Article in Portuguese] doi: 10.36660/abc.20200162 (PMC9744355; doi:10.36660/abc.20200162)
Supplement: Translation

# Translation: Inspiratory Muscle Training at Different Intensities in Heart Failure: Are There Differences in Central Hemodynamic Changes?

**Authors:** Lucas Helal, Filipe Ferrari

**Affiliations:** 1 Brazil Universidade do Extremo Sul Catarinense - UNESC, Criciúma, SC - Brazil; 2 Brazil Graduate Program in Cardiology and Cardiovascular Sciences, Hospital de Clínicas de Porto Alegre (HCPA), Universidade Federal do Rio Grande do Sul, Porto Alegre, RS - Brazil; 3 Canada Centre for Journalology, Ottawa Hospital Research Institute, Ottawa - Canada; 4 Brazil Exercise Cardiology Research Group (CardioEx), Hospital de Clínicas de Porto Alegre, Universidade Federal do Rio Grande do Sul, Porto Alegre, RS - Brazil

**Keywords:** Heart Failure, Maximal Respiratory Pressures, Heart Rate, Stroke Volume, Cardiac Output, Hemodynamic Monitoring/methods

Heart failure (HF) is a complex entity and usually has a poor prognosis, identified as an extremely relevant cardiovascular disease due to its increasing incidence, prevalence and high associated morbidity and mortality.^1^It is estimated that its prevalence varies from 1% to 2% in developed countries, reaching >10% in people over 70 years of age;^2^moreover, it is considered the main cause of cardiovascular hospitalization in individuals older than 60 years.^3^

Among the main symptoms characteristic of HF, dyspnea and fatigue stand out, which are closely associated with impaired functional capacity and, consequently, with these individuals’ quality of life.^4^Physical training, in turn, has gained a prominent place in recent decades, aiming at improving this scenario, based on increasing evidence; therefore, it has the ability to favorably impact the gain in functional capacity and quality of life of HF patients.^5 , 6^Among the several types of physical training targeted at this population, inspiratory muscle training (IMT) is known for being easy to apply and showing potential benefits in this scenario.

## Inspiratory muscle training in heart failure

There is robust evidence suggesting that the weakness of inspiratory muscles is one of the main factors that lead to low exercise tolerance in patients with HF.^7 , 8^In fact, randomized clinical trials have shown several benefits of IMT in patients with this syndrome, namely: significant improvement in oxygen uptake efficiency,^9^functional capacity and quality of life scores.^10^These results were confirmed by meta-analyses, such as that carried out by Smart et al.^11^When compared to the control group, patients undergoing IMT achieved an important improvement in maximum oxygen consumption (VO_2_max): 1.83 mL.kg^-1^.min^-1^(95%CI, 1.33 for 2.32 mL.kg^-1^.min^-1^, p <0.00001), as well as in the 6-minute walking test: 34.35 m (95%CI, 22.45 to 46.24 m, p <0.00001). In turn, inspiratory muscle strength seems to have a significant correlation with VO_2_max, which is an independent predictor of survival in individuals with HF.^12^IMT should therefore be an integral part of these patients’ care whenever possible.

In this issue of the *Arquivos Brasileiros de Cardiologia* , a randomized, placebo-controlled trial^13^evaluated the implications of an acute session of different intensities of IMT on the central hemodynamic response (CHR) of individuals with HF, using a non-invasive monitoring method. For this purpose, 20 patients with reduced ejection fraction (37.2% ± 6.3%), a mean age of 65 years, and the vast majority in New York Heart Association (NYHA) functional class II were included in the study. The IMT protocol consisted of 3 sessions lasting 15 minutes each. All participants underwent the training with an intensity of 30% and 60% of maximum inspiratory pressure (MIP), in addition to sham intervention (placebo), with a 1-hour washout between them. It was observed that CHR behaved in a heterogeneous way between intensities. For instance, there was an increase in heart rate with intensities of 30% and 60% of MIP (64 ± 15 to 69 ± 15 beats per minute; and 67 ± 14 to 73 ± 14 beats per minute, respectively). Regarding stroke volume, there was a tendency to decrease with a 30% load of MIP (73 ± 26 mL to 64 ± 20 mL). The cardiac output increased only in the group with the highest intensity (4.6 ± 1.5 L / min to 5.3 ± 1.7 L / min), a behavior that was similar in relation to the systolic blood pressure response. In fact, the increase in cardiac output observed at the highest applied intensity can be partially explained by the increase in heart rate in this group. These findings should not be ignored, since patients with HF tend to have impaired blood flow to the active muscles, secondary to reductions in cardiac output and peripheral vasodilator capacity. These changes are harmful, causing important intolerance to effort, being associated with reduced vasodilator capacity and increased sympathetic stimulation, common in these individuals.^14^

Recently, in a systematic review with meta-analysis, our group showed that high-intensity IMT (≥ 60% of MIP) can be an efficient strategy to improve functional capacity and inspiratory muscle strength in this same class of patients (that is: HF and reduced ejection fraction).^15^In turn, we believe that the referred study^13^adds important knowledge to the literature, showing differences in the hemodynamic repercussions observed in the IMT at different intensities, an area little explored so far. These findings may open new horizons and perspectives, influencing further research exploring the hemodynamic responses of IMT in patients with HF. The lack of a close correlation between central hemodynamics (such as the sympathetic nervous system hyperactivation) and exercise tolerance strengthens the importance of the results of this study.
